# Impact of Nodular Calcifications in the Aortic Annulus and Left Ventricular Outflow Tract on TAVI Outcome with New-Generation Devices

**DOI:** 10.31083/j.rcm2311358

**Published:** 2022-10-25

**Authors:** Riccardo Gorla, Omar A. Oliva, Enrico Poletti, Alice Finotello, Simone Morganti, Jessica Zannoni, Mauro Agnifili, Marta Barletta, Mattia Squillace, Enrico Criscione, Maurizio Tusa, Nedy Brambilla, Ferdinando Auricchio, Luca Testa, Francesco Bedogni

**Affiliations:** ^1^Department of Clinical and Interventional Cardiology, IRCCS Policlinico San Donato, 20097 San Donato Milanese (MI), Italy; ^2^Department of Civil Engineering and Architecture, University of Pavia, 27100 Pavia, Italy; ^3^Department of Electrical, Computer and Biomedical Engineering, University of Pavia, 27100 Pavia, Italy

**Keywords:** transcatheter aortic valve implantation, nodular calcification, device success, paravalvular leak, computational simulation

## Abstract

**Background:**

The impact of nodular calcifications in left ventricular outlow tract (LVOT) and aortic annulus on the procedural outcome of transcatheter aortic valve implantation (TAVI) with new-generation devices is yet to be elucidated. Similarly, computational simulations may provide a novel insight into the biomechanical features of TAVI devices and their interaction with nodular calcifications.

**Methods:**

This retrospective single-center study included 232 patients submitted to TAVI with Evolut-R (53.4%), Portico (33.6%) and Lotus (13.0%) devices with available preoperative computed tomography (CT) angiography and evidence of nodular calcifications in aortic annulus and/or LVOT. Calcification severity was defined ≥moderate in presence of at least two nodules or one nodule ≤5 mm. Three virtual simulation models of aortic root presenting a nodular calcification of increasing size were implemented. Stress distribution, stent-root contact area and paravalvular orifice area were computed.

**Results:**

At least moderate calcifications were found in 123 (53.0%) patients, with no sex differences. Among the ≥moderate calcification group, lower device success rate was evident (87.8% vs. 95.4%; *p* = 0.039). Higher rates of ≥moderate paravalvular leak (PVL) (11.4% vs. 3.7%; *p* = 0.028) and vascular complications (9.8% vs. 2.8%; *p* = 0.030) were also observed. Among the Evolut-R group, higher rates of at ≥moderate PVL (12.1%) were observed compared to Portico (3.8%; *p* = 0.045) and Lotus (0.0%; *p* = 0.044) groups. Calcification of both annulus and LVOT (odds ratio [OR] 0.105; *p* = 0.023) were independent predictors of device success. On computational simulations, Portico exhibited homogeneous stress distribution by increasing calfications and overall a larger paravalvular orifice areas compared to Evolut-R and Lotus. Evolut-R showed higher values of average stress than Portico, although with a more dishomogeneous distribution leading to greater paravalvular orifice areas by severe calcifications. Lotus showed overall small paravalvular orifice areas, with no significant increase across the three models.

**Conclusions:**

At least moderate nodular calcifications in the annulus/LVOT region significantly affected TAVI outcome, as they were independent predictors of device success. Lotus and Portico seemed to perform better than Evolut-R as for device success and ≥moderate PVL. Computational simulations revealed unique biomechanical features of the investigated devices in terms of stent compliance and radial force.

## 1. Introduction

Transcatheter aortic valve implantation (TAVI) is an established treatment option for patients with symptomatic severe aortic stenosis at high and intermediate surgical risk [1, 2], with comparable results to surgery also in low risk patients [3, 4].

Both Evolut-R (Medtronic Inc., Minneapolis, MN, USA) and Portico aortic valves (Abbott, Minneapolis, MN, USA) are self-expanding (SE) devices [5, 6]. The Lotus valve (Boston Scientific, Marlborough, MA, USA) is the only mechanically expandable (ME) device, and, as unique feature, allows full retrievability even after complete deployment [7].

Device success depends on several features, which are related either to the aortic root and valve anatomy (e.g., calcifications, aortic angulation) and technical aspects (e.g., oversizing, implantation depth) [8, 9, 10, 11]. One of the anatomical factors that may be relevant to procedural success is the size and dimension of the annular and left ventricular outflow tract (LVOT) calcifications [8]. Prosthetic valves are meant to be expanded in a circular fashion, hence the presence of nodular calcifications may lead to partial underexpansion of the strut, with consequent paravalvular leak (PVL) [12].

Thus, the strut conformability of new-generation SE and ME devices may be relevant. In this setting, computational simulations may constitute a compelling tool to predict the device performance, based on its interaction with the aortic annulus geometry altered by nodular calcifications [13].

Aim of the present study is to assess the impact of nodular calcifications in the aortic annulus and LVOT on device success and residual PVL in patients undergoing TAVI with new-generation devices and to test by computational simulations their biomechanical behaviour with respect to the severity of nodular calcification.

## 2. Materials and Methods

### 2.1 Study Design and Data Collection

This retrospective, observational, single-center study included patients with symptomatic severe aortic stenosis undergoing TAVI with Evolut-R, Portico and Lotus devices at the IRCCS Policlinico San Donato from January 2016 to May 2021 with available computed tomography (CT) angiography aortic annulus measurements.

Inclusion criterion was the presence of discrete nodular annular and/or LVOT calcifications.

Exclusion criteria were pure aortic regurgitation as indication for TAVI, and valve-in-valve TAVI. Patients treated with Evolut Pro were excluded from the analysis; merging these patients with those treated with Evolut-R could have biased the results due to the external sealing skirt in the formers. Additionally, computational simulations could only consider the prosthetic valve stent.

Among 687 patients treated with Evolut-R, Portico or Lotus valve, 232 (33.8%) were included in the study population.

Our institutional TAVI database collected prospectively the data about baseline features, procedural aspects, echocardiography measures, CT scan and 30-days outcomes.

All patients proceeded to TAVI after Heart Team discussion and provided written informed consent before the procedure.

### 2.2 CT-Angiography and Aortic Nodular Calcification Measurement

**Fig. 1.**
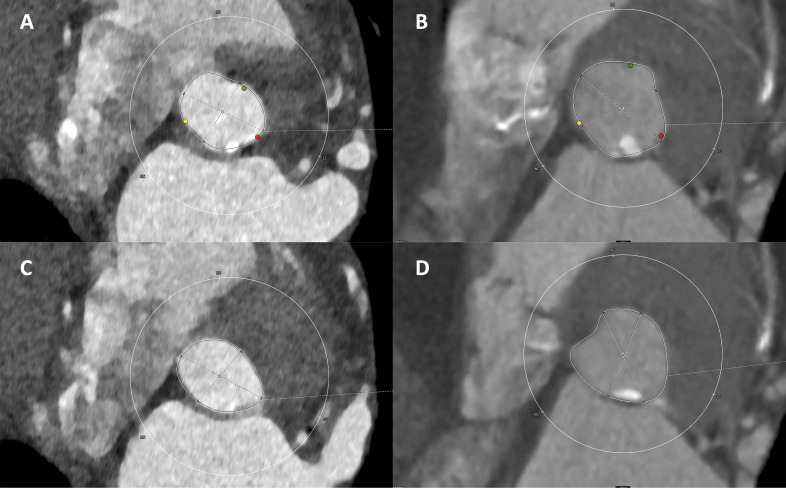
**Evaluation of calcification severity at CT angiography**. Mild calcification severity: a calcified nodule (max diameter 2.25 mm) in the annulus (A); LVOT is free from calcifications (C); ≥moderate calcification severity: two nodular calcifications with a max diameter of 7.5 mm and of 6 mm respectively are present at the annulus (B) and LVOT (D).

CT-angiography scans were performed on 64- or 128-row multidetector scanner (Somatom Definition; Siemens healthcare, Forchheim, Germany). Image acquisition was electrocardiography (ECG) gated. The 3-Mensio valves software (version 8.2, Pie Medical Imaging, Maastricht, The Netherlands) permitted the multiplanar reconstruction analysis of the aortic root, evaluating both the diastolic and systolic phase [14]. Calcification severity was defined mild in presence of one spherical, calcific nodule with a major diameter <5 mm and ≥moderate in presence of at least two nodules or one nodule ≥5 mm [15]. The nodules could be either in the aortic annulus or in the LVOT (Fig. 1).

It was also taken into account the index of eccentricity (IE), calculated as Eqn. 1:

(1) IE=(1- short axis / long axis annulus diameter )

IE >0.25 defined an elliptic aortic annulus [16]. Oversizing was determined as Eqn. 2:

(2) $$\begin{array}{c} \text{ calculated perimeter oversizing }\left(\%\right)=\\ \left(\text{ prosthesis }/\text{ annulus perimeter }-1\right)*100. \end{array}$$

Areas of calcium were detected in the region of interest (from the virtual basal ring up to 4 mm in the LVOT) using a validated threshold of 800 Hounsfield Units (HU) [17]. A dedicated core laboratory of radiology technicians made all measurements. They were blinded to the implanted prosthesis before TAVI.

### 2.3 Transcatheter Aortic Valve Implantation

Transfemoral TAVI was performed under local anesthesia with or without conscious sedation according to patient’s tolerance to the procedure; trans-subclavian and transaortic TAVI were performed under general anesthesia [18].

Technical details of the Evolut-R, Portico and Lotus device have been previously reported [5, 6, 7].

Due to our internal policy, Evolut-R was the most employed prosthesis at our Institution. In light of this consideration, the device was used in about half procedures (124/232; 53.4%), followed by Portico (78/232; 33.6%) and by Lotus (30/232; 12.9%). Given this premise, in each case, prosthesis choice was left to first operator’s discretion.

Implantation depth was defined as the maximal distance between the bioprosthetic intraventricular edge and the aortic annulus at the level of the non-coronary cusp (NCC) and left coronary cusp (LCC), measured by angiography in the deployment projection [19].

Repositioning was defined as partial valve resheathing to enable movement from its initial deployment site forward or backward in the ventricle. Recapture was defined as complete valve retraction into the delivery catheter [20].

### 2.4 Transthoracic Echocardiography

Transthoracic echocardiography (TTE) was performed with a GE Vivid 9 ultrasound unit (GE Healthcare, Horten, Norway) before and after TAVI. Postprocedural TTE was performed the same day of procedure and repeated at discharge. Post-procedural PVL was assessed in line with Valve Academic Research Consortium-3 (VARC-3) criteria and classified in four categories (absent/trivial, mild, moderate, severe) by experienced echocardiographers [21].

An independent reader blinded both to aortic annulus measurements and to prosthesis type manually reviewed the cine-loops; discrepancies in PVL grading were resolved by consensus. Moreover, discharge TTE data were used for the analysis in case of discrepancies with the post-procedural ones, as the self-expanding mechanism of steel may contribute to improve PVL severity during periprocedural period. At last, trivial jets were grouped with no PVL, whereas moderate and severe PVL were grouped together as ≥moderate PVL.

### 2.5 Device Success

Device success was defined according to VARC-3 definition upon fulfilling the following criteria: (1) absence of procedural mortality (within 72 h from the procedure); (2) correct positioning of a single prosthetic transcatheter heart valve (THV) into the proper anatomical location; (3) intended prosthetic THV performance (no patient-prosthesis mismatch, mean aortic gradient <20 mmHg and absence of ≥moderate PVL) [21].

### 2.6 Simulation Framework

Evolut-R, Portico and Lotus were compared. Geometrical models were reconstructed from micro-CT scans of real device samples (Evolut-R 26 mm, Portico 25 mm, and Lotus Edge 23 mm) and Nitinol material properties were assigned [22]. Only the prosthetic valve stent was considered since the valve was not visible from CT images and the post-operative configurations of the stent and aortic root were assumed not to be influenced by prosthetic leaflets.

**Fig. 2.**
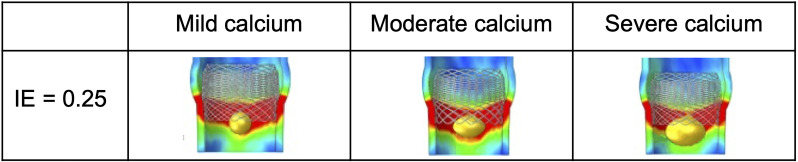
**Nodular calcification model**. Example of TAVI simulation with Lotus valve in a 0.25 IE aortic root having nodular calcifications of increasing size (maximum diameter: mild 8 mm, moderate 12 mm, severe 14 mm).

An idealized aortic root model composed of three regions (annulus/LVOT, valsalva sinuses and ascending aorta) was conceived as previously reported [10]. Only the aortic root was considered, native leaflets were not taken into account in the simulation framework. In a previous study the impact of an elliptic vs. circular annulus has been evaluated [10]. Moreover, as in the present study the IE was 0.21 ± 0.07; the simulations in a non-circular aortic root model with an IE 0.25 were performed, as it could be real-world like (diameters of aortic model: annulus and LVOT 25 mm × 18.8 mm, Valsalva sinuses 32 mm, sino-tubular junction 28 mm). Three different sizes of nodular calcium were stratified by increasing severity (mild, moderate, and severe). The calcifications were modeled as a portion of sphere or ellipsoid leaning on the aortic root (Fig. 2).

Material properties to model the arterial soft tissue and calcifications were derived from a previous publication [23].

These idealized aortic root models were used as the initial geometries for finite element simulation of TAVI using the commercial software Abaqus (v. 2019, Simulia, Dàssault Systems, Providence, RI, USA). The simulation setup consisted of two steps: crimping of the stent inside its catheter and stent re-expansion within the aortic root models with a final implantation depth according to each device’s instructions by the manifacturer. More details about the simulation procedure were given in a previous publication of our research group [24]. Post-processing of simulation outcomes was then performed and three variables were measured: the stent-root interaction area, Von Mises stress distribution, and paravalvular orifice area [24].

**Fig. 3.**
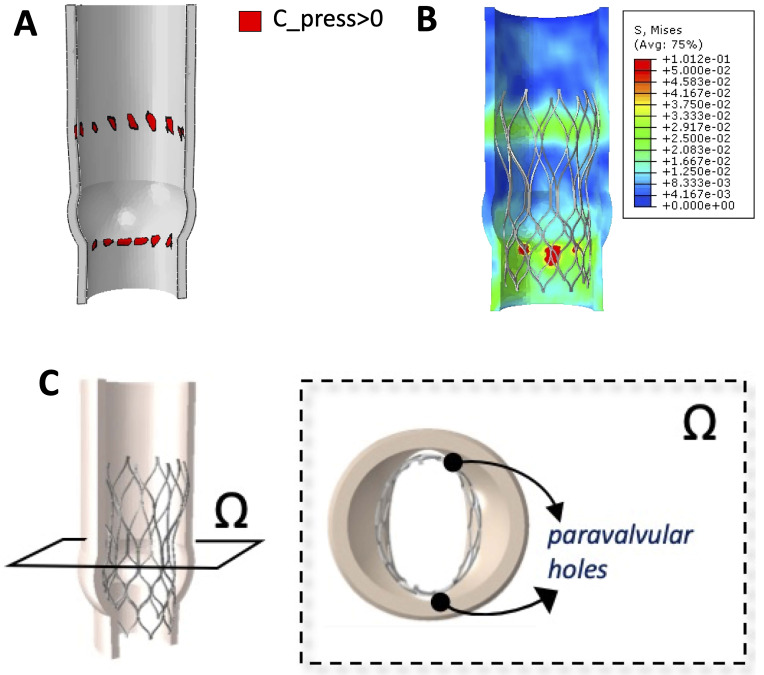
**Stent-rott interaction model**. (A) Stent-root interaction area: contact area between the stent and the internal surface of the aortic root. (B) Von Mises stress map: distribution of stress values in the inner aortic root. (C) Paravalvular orifice area: definition of a cutting plane Ω passing through the sino-tubular junction and cross-section of the model at the level of the plane to identify paravalvular orifices.

The measure of the stent-root interaction area could represent an indication of stent anchoring and adesion to the wall. It was computed by means of an in-house Matlab script (The MathWorks, Inc., Natick, MA, USA) as the sum of the areas of the aortic wall elements with contact pressure higher than zero (Fig. 3A).

Von Mises stress distribution is a measure of the stress induced by the device expansion onto the inner wall of the aortic root (Fig. 3B). Only the annulus/LVOT internal wall region was considered. Both the average stress and the maximum one were computed with a Matlab script. To make the interpretation of potential dishomogeneity in the stress distribution clearer, the ratio between the average and the maximum stress value was shown (low values meant a more dishomogeneous stress distribution against the aortic wall). Paravalvular orifice area was derived from the area of the orifices generated after stent expansion between the device and the inner aortic root wall. The area of the such orifices was quantified using the open source software Image J (1.52t (30 January 2020), JAVA, NIH, USA) [24] (Fig. 3C).

### 2.7 Statistical Analysis

Categorical and dichotomous variables are shown as frequencies and percentages; they were compared by Pear- son chi-square or Fisher exact tests, as appropriate.

The Kruskal-Wallis test was used to check the skewed distribution of continuous covariates.

Continuous variables following a normal distribution are reported as mean and standard deviation; they were compared using unpaired two-sided Student’s *t*-test. Otherwise, non-normally distributed variables were arranged as median and interquartile range; they were compared using the Mann-Whitney U-test.

Univariate logistic regression analysis was performed to investigate factors associated with ≥moderate PVL.

Multivariate logistic regression analysis was performed to investigate factors associated with device success, by using a backward stepwise method including variables with *p *< 0.20 on univariate analysis.

All *p*-values were two-sided with values <0.05 considered statistically significant. Analyses were performed using SPSS 27.0 statistical analysis software (IBM Corporation, Armonk, NY, USA).

## 3. Results

### 3.1 Baseline Characteristics

Among the study population, 123 (53.0%) patients showed ≥moderate calcifications on preoperative CT-angiography.

Patients with mild calcifications had more frequently a history of prior coronary artery bypass grafting (CABG). There were no significant differences regarding age, other cardiovascular risk factors, Society of Thoracic Surgeons (STS) score, creatinine clearance, and echocardiographic findings.

On CT-angiography, patients with ≥moderate calcifications showed higher Calcium volume 800 HU, and higher indexes of calcification. Annulus perimeter, Valsalva sinus diameter, IE and coronary artery height were similar among the two groups (Table 1).

**Table 1.** **Baseline characteristics based on calcification severity**.

Variables	Overall calcium	Mild calcifications	≥Moderate calcifications	*p*-value
N	232 (100%)	109 (47.0%)	123 (53.0%)	
Age (years)	83.0 ± 7.3	82.1 ± 7.8	83.7 ± 6.7	0.325
Female sex	131 (56.5%)	63 (57.8%)	68 (55.3%)	0.700
Hypertension	169 (72.8%)	80 (73.4%)	89 (72.4%)	0.859
Diabetes	55 (23.7%)	27 (24.8%)	28 (22.8%)	0.720
Dyslipidemia	92 (39.7%)	42 (38.5%)	50 (40.7%)	0.742
COPD	35 (15.1%)	19 (17.4%)	16 (13.0%)	0.348
Coronary artery disease	76 (32.8%)	33 (30.3%)	43 (35.0%)	0.448
Prior CABG	17 (7.4%)	13 (12.0%)	4 (3.3%)	0.011
Prior AMI	26 (11.2%)	13 (11.9%)	13 (10.6%)	0.744
STS score (%)	5.0 ± 3.0	5.2 ± 3.2	4.8 ± 2.6	0.197
Creatinine clearance (mL/min/1.73 $m^{2}$)	53.0 ± 21.1	50.0 ± 22.6	55.8 ± 19.1	0.086
Haemoglobin (g/dL)	12.0 ± 1.7	12.0 ± 1.7	12.0 ± 1.6	0.180
Ejection fraction (%)	55.5 ± 11.4	54.5 ± 11.9	56.6 ± 10.9	0.619
Mean aortic gradient (mmHg)	46.0 ± 15.9	44.82 ± 15.5	47.23 ± 16.3	0.649
AR ≥moderate	48 (20.7%)	20 (18.3%)	28 (22.8%)	0.407
LM height (mm)	14.7 ± 3.8	14.5 ± 4.2	14.9 ± 3.5	0.300
RCA height (mm)	17.8 ± 3.7	17.6 ± 3.6	17.9 ± 3.8	0.862
Annulus min diameter (mm)	20.8 ± 2.6	20.6 ± 2.5	20.9 ± 2.6	0.907
Annulus max diameter (mm)	26.5 ± 2.9	26.4 ± 2.9	26.5 ± 2.9	0.965
Annulus mean diameter (mm)	23.6 ± 2.6	23.5 ± 2.6	23.6 ± 2.6	0.863
Annulus perimeter (mm)	74.8 ± 7.9	74.6 ± 8.0	75.1 ± 7.8	0.794
Annulus area (${\mathrm{mm}}^{2}$)	415.2 ± 120.8	397.2 ± 138.2	431.2 ± 100.8	0.087
Sinus of Valsalva diameter (mm)	32.4 ± 4.2	31.9 ± 4.3	32.7 ± 4.0	0.398
Calcium volume 800 HU (${\mathrm{mm}}^{3}$)	304.2 [175.8–549.9]	236.5 [151.6–437.9]	416.3 [216.4–654.8]	<0.001
Aortic angulation (°)	49.9 ± 10.9	49.3 ± 11.5	50.4 ± 10.3	0.206
Index of eccentricity	0.21 ± 0.07	0.22 ± 0.06	0.21 ± 0.07	0.994
Calcification in LVOT	134 (57.8%)	45 (41.3%)	89 (72.4%)	<0.001
Calcification at annulus	188 (81.0%)	78 (71.6%)	110 (89.4%)	<0.001
Calcification at annulus and LVOT	90 (38.8%)	14 (12.8%)	76 (61.8%)	<0.001
Calcification number	1.42 ± 0.7	1.0 ± 0.0	1.79 ± 0.71	<0.001
Major calcification diameter (mm)	4.6 ± 2.7	2.9 ± 1.0	6.1 ± 2.8	<0.001
LVOT diameter (mm)	19.3 ± 2.9	19.4 ± 2.8	19.1 ± 2.9	0.884
Ascending aorta (mm)	34.4 ± 4.8	34.0 ± 4.5	34.8 ± 5.1	0.980

AMI, acute myocardial infarction; AR, aortic regurgitation; CABG, coronary artery bypass grafting; COPD, chronic obstructive pulmonary disease; HU, Hounsfield Units; LM, left main; LVOT, left ventricular outflow tract; RCA, right coronary artery; STS, Society of Thoracic Surgeons.

Patients treated with Lotus valve were younger, had lower STS score and smaller major diameter calcifications rather than Evolut-R and Portico patients. Evolut-R patients had a higher prevalence of diabetes, meanwhile Portico patients had lower levels of haemoglobin and lower values of annulus diameter (mean and maximum).

On CT-angiography, Portico treated patients had lower coronary artery take-off and annulus perimeter.

No significant differences in the rate of ≥moderate calcification were noted among the devices (52.4% for Evolut-R, 59.0% for Portico and 40% for Lotus, *p* = 0.205), whereas the rates of calcification location differed significantly (Table 2).

**Table 2.** **Baseline characteristics of Evolut-R, Portico and Lotus patients**.

Variables	Evolut-R	Portico	Lotus	*p*-value
N	124 (53.4%)	78 (33.6%)	30 (12.9%)	
Age (years)	86.6 ± 6.7	83.8 ± 7.1	78.0 ± 8.2	<0.001
Female sex	68 (54.8%)	49 (62.8%)	14 (46.7%)	0.274
Hypertension	85 (68.5%)	61 (78.2%)	23 (76.7%)	0.285
Diabetes	38 (30.6%)	14 (17.9%)	3 (10.0%)	0.020
Dyslipidemia	43 (34.7%)	39 (50.0%)	10 (33.3%)	0.072
COPD	19 (15.3%)	11 (4.7%)	5 (15.7%)	0.940
Coronary artery disease	42 (33.9%)	21 (26.9%)	13 (43.3%)	0.247
Prior CABG	9 (7.3%)	5 (6.4%)	3 (10.0%)	0.785
Prior AMI	13 (10.5%)	9 (11.5%)	4 (13.3%)	0.900
STS score (%)	5.4 ± 3.2	5.0 ± 2.9	3.4 ± 2.0	0.005
Creatinine clearance (mL/min/1.73 $m^{2}$)	51.9 ± 21.1	52.0 ± 22.2	59.1 ± 17.8	0.226
Haemoglobin (g/dL)	12.1 ± 1.7	11.6 ± 1.5	12.7 ± 1.7	0.018
Ejection fraction (%)	55.2 ± 11.9	55.9 ± 11.2	55.7 ± 10.2	0.932
Mean aortic gradient (mmHg)	46.4 ± 16.1	43.5 ± 14.3	50.8 ± 17.9	0.109
AR ≥moderate	27 (21.8%)	15 (19.2%)	6 (20.0%)	0.905
LM height (mm)	15.1 ± 3.8	13.8 ± 3.6	15.5 ± 4.1	0.050
RCA height (mm)	18.3 ± 3.7	16.8 ± 3.6	18.1 ± 3.5	0.015
Annulus min diameter (mm)	20.9 ± 2.8	20.3 ± 2.2	21.6 ± 2.2	0.066
Annulus max diameter (mm)	26.8 ± 3.1	25.6 ± 2.7	27.2 ± 2.2	0.004
Annulus mean diameter (mm)	23.8 ± 2.8	23.0 ± 2.3	24.4 ± 2.0	0.014
Annulus perimeter (mm)	75.6 ± 8.5	72.6 ± 6.9	77.6 ± 6.1	0.004
Annulus area (${\mathrm{mm}}^{2}$)	438.5 ± 101.1	396.7 ± 101.1	366.0 ± 201.9	0.003
Sinus of Valsalva diameter (mm)	32.7 ± 4.3	31.2 ± 3.9	32.7 ± 4.4	0.162
Calcium volume 800 HU (${\mathrm{mm}}^{3}$)	299. 4 [172.2–526.5]	276.5 [169.3–479.8]	519.2 [204.7–802.2]	0.187
Aortic angulation (°)	50.9 ± 11.3	47.9 ± 9.5	50.9 ± 10.9	0.164
Index of eccentricity	0.22 ± 0.07	0.20 ± 0.07	0.21 ± 0.05	0.163
Calcification at LVOT	71 (57.3%)	52 (66.7%)	11 (36.7%)	0.018
Calcification at annulus	105 (84.7%)	56 (71.8%)	27 (90%)	0.031
Calcification in annulus and LVOT	52 (41.9%)	30 (38.5%)	8 (26.7%)	0.305
Calcification number	1.36 ± 0.72	1.51 ± 0.78	1.40 ± 0.72	0.378
Major calcification diameter (mm)	4.5 ± 2.4	5.2 ± 3.0	3.7 ± 2.4	0.024
LVOT diameter (mm)	65 (52.4%)	46 (59.0%)	12 (40.0%)	0.205
Ascending aorta (mm)	19.3 ± 2.8	18.9 ± 2.3	20.5 ± 4.3	0.109

AMI, acute myocardial infarction; AR, aortic regurgitation; CABG, coronary artery bypass grafting; COPD, chronic obstructive pulmonary disease; HU, Hounsfield Units; LM, left main; LVOT, left ventricular outflow tract; RCA, right coronary artery; STS, Society of Thoracic Surgeons.

### 3.2 Procedural Data

TAVI was performed through femoral access in 91.4% of procedures; among the ≥moderate calcifications group there was a higher rate of any vascular complications (9.8% vs. 2.8%, *p* = 0.030) and higher radiaton doses (86.5 ± 62.1 ${\mathrm{Gycm}}^{2}$ vs. 72.8 ± 48.9 ${\mathrm{Gycm}}^{2}$, *p* = 0.029), an indirect marker of longer procedural time. No differences were evident regarding predilation, postdilatation, implantation depth, stenting of the access site and concomitant percutaneous coronary intervention (PCI) (Table 3).

**Table 3.** **Procedural data based on calcification severity**.

Variables	Overall calcium	Mild calcifications	≥Moderate calcifications	*p*-value
N	232 (100%)	109 (47%)	123 (53%)	
Femoral route	212 (91.4%)	102 (93.6%)	110 (89.4%)	0.261
Subclavian route	20 (8.6%)	7 (6.4%)	13 (10.6%)	0.261
Embolic protection system	13 (5.6%)	7 (6.4%)	6 (4.9%)	0.610
Any vascular complications	15 (6.5%)	3 (2.8%)	12 (9.8%)	0.030
PTA with stenting of access site	4 (1.7%)	1 (0.9%)	3 (2.4%)	0.625
PCI with stenting	12 (5.2%)	5 (4.6%)	7 (5.7%)	0.705
Degree of oversizing (%)	15.9 ± 9.5	15.9 ± 10.4	15.8 ± 8.6	0.060
Predilatation	131 (56.5%)	58 (44.3%)	73 (55.7%)	0.347
Implantation depth NCC (mm)	4.4 ± 2.6	4.6 ± 2.4	4.2 ± 2.8	0.323
Implantation depth LCC (mm)	5.9 ± 3.0	6.2 ± 3.0	5.7 ± 3.1	0.585
Implantation depth mean (mm)	5.1 ± 2.6	5.4 ± 2.5	5.0 ± 2.8	0.500
Postdilatation	122 (52.6%)	58 (54.2%)	64 (52.5%)	0.858
Repositioning	55 (23.7)	25 (22.9%)	30 (24.4%)	0.795
Recapture	47 (20.3%)	21 (19.3%)	26 (21.1%)	0.723
Emergent cardiac surgery	1 (0.04%)	0 (0.0%)	1 (0.8%)	1.000
Need for second valve	1 (0.04%)	0 (0.0%)	1 (0.8%)	1.000
Contrast volume (mL)	155.3 ± 62.6	146.1 ± 52.4	164.6 ± 70.6	0.101
Radiation dose (${\mathrm{Gycm}}^{2}$)	80.1 ± 56.6	72.8 ± 48.9	86.5 ± 62.1	0.029

LCC, left coronary cusp; NCC, non-coronary cusp; PCI, percutaneous coronary intervention; PTA, percutaneous transluminal angioplasty.

Postdilatation rate was significantly lower in the Lotus group compared to Evolut-R and Portico, whereas the Evolut group had the lowest rate of predilatation. Implantation depth was greater in Portico valve patients. Degree of oversizing, as expected, was significantly different among the devices, due to the sizing chart of each valve. Similarly, Lotus valve, due to its unique feature, was the most repositioned device.

No differences were noticed regarding recapture rates, any vascular complications, radiation dose and concomitant PCI (Table 4).

**Table 4.** **Procedural data of Evolut-R, Portico and Lotus patients**.

Variables	Evolut-R	Portico	Lotus	*p*-value
N	124 (53.4%)	78 (33.6%)	30 (12.9%)	
Femoral route	113 (91.1%)	69 (88.5%)	30 (100%)	0.159
Subclavian route	11 (8.9%)	9 (11.5%)	0 (0.0%)	0.159
Embolic protection system	8 (6.5%)	2 (2.6%)	3 (10.0%)	0.269
Any vascular complications	8 (6.5%)	6 (7.7%)	1 (3.3%)	0.711
PTA with stenting of access site	1 (0.8%)	2 (2.6%)	1 (3.3%)	0.497
PCI with stenting	6 (4.8%)	6 (7.7%)	0 (0.0%)	0.263
Degree of oversizing (%)	20.8 ± 6.8	13.8 ± 6.8	1.1 ± 7.7	<0.001
Predilatation	52 (41.9%)	60 (76.9%)	19 (63.3%)	<0.001
Implantation depth NCC (mm)	4.3 ± 2.5	4.9 ± 2.7	3.4 ± 2.6	0.047
Implantation depth LCC (mm)	5.6 ± 3.0	6.8 ± 2.9	5.0 ± 3.1	0.012
Implantation depth mean (mm)	5.0 ± 2.6	5.8 ± 2.7	5.1 ± 2.6	0.015
Postdilatation	72 (58.1%)	48 (61.5%)	2 (6.7%)	<0.001
Repositioning	15 (12.1%)	26 (3.3%)	14 (46.7%)	<0.001
Recapture	27 (21.8%)	12 (15.4%)	8 (26.7%)	0.352
Emergent cardiac surgery	1 (0.08%)	0 (0.0%)	0 (0.0%)	0.646
Need for second valve	0 (0.0%)	1 (1.3%)	0 (0.0%)	0.371
Contrast volume (mL)	151.9 ± 63.0	167.2 ± 67.6	137.9 ± 37.3	0.132
Radiation dose (${\mathrm{Gycm}}^{2}$)	76.1 ± 51.5	81.0 ± 62.2	106.8 ± 55.2	0.182

LCC, left coronary cusp; NCC, non-coronary cusp; PCI, percutaneous coronary intervention; PTA, percutaneous transluminal angioplasty.

### 3.3 In-Hospital Outcome

There were no significant differences regarding permanent pacemaker implantation rate, 30-day mortality and stroke among the two groups based on calcifications severity. The ≥moderate calcifications group showed on TTE a higher rate of ≥moderate PVL (11.4% vs. 3.7%, *p* = 0.028). Also the device success rate was lower (87.8% vs. 95.4%, *p* = 0.039) (Table 5).

**Table 5.** **In-hospital outcome based on calcification severity**.

Variables	Overall calcium	Mild calcifications	≥Moderate calcifications	*p*-value
N	232 (100%)	109 (47.0%)	123 (53.0%)	
Ejection fraction (%)	57.2 ± 10.2	55.5 ± 11.0	58.8 ± 9.2	0.760
Mean gradient (mmHg)	9.7 ± 4.1	9.6 ± 4.4	9.7 ± 3.9	0.353
PVL absent/trivial	82 (35.3%)	41 (37.6%)	41 (33.3%)	0.496
PVL mild	132 (56.9%)	64 (58.7%)	68 (55.3%)	0.598
PVL ≥moderate	18 (7.8%)	4 (3.7%)	14 (11.4%)	0.028
Device success	212 (91.4%)	104 (95.4%)	108 (87.8%)	0.039
PPI	37 (15.9%)	14 (12.8%)	23 (18.7%)	0.224
Stroke (including not disabling)	5 (2.2%)	3 (2.8%)	2 (1.6%)	0.555
30-day mortality	10 (4.3%)	4 (3.7%)	6 (4.9%)	0.753

PPI, permanent pacemaker implantation; PVL, paravalvular leak.

**Fig. 4.**
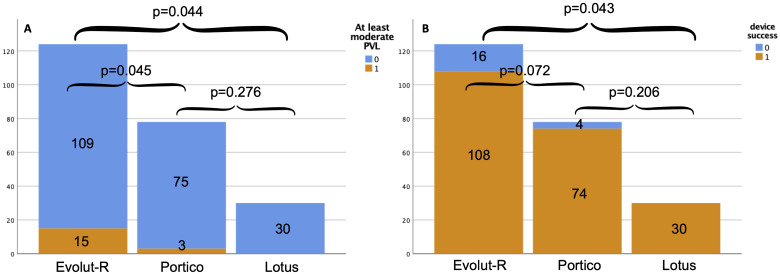
**In-hospital outcomes**. Differences between Evolut-R, Portico and Lotus valves groups by ≥moderate PVL (A) and by device success (B) rate. Higher rates of ≥moderate PVL were observed in the Evolut-R group, compared both to Portico and Lotus patients (A). On the other hand, Evolut-R group showed lower device success rates compared to the Lotus group (*p* = 0.043), but did not reach statistical significancy with the Portico group (*p* = 0.072) (B).

When stratified by prosthesis type (Table 6), higher rates of ≥moderate PVL were observed in the Evolut-R group, compared both to Portico and Lotus groups (Fig. 4A). Additionally, the Evolut-R group showed a lower device success rate compared to the Lotus group, but did not reached the statistical significancy with the Portico one (Fig. 4B). Lotus valves showed a statistically significant higher transprosthetic mean gradient compared to Evolut-R and Portico.

**Table 6.** **In-hospital outcome of Evolut-R, Portico and Lotus patients**.

Variables	Evolut-R	Portico	Lotus	*p*-value
N	124 (53.4%)	78 (33.6%)	30 (12.9%)	
Ejection fraction (%)	56.7 ± 10.8	58.1 ± 9.8	56.7 ± 10.2	0.729
Mean gradient (mmHg)	8.7 ± 3.7	9.2 ± 4.3	12.3 ± 3.6	<0.001
PVL absent/trivial	36 (29.0%)	25 (32.1%)	21 (70.0%)	<0.001
PVL mild	73 (58.9%)	50 (64.1%)	9 (30%)	0.005
PVL ≥moderate	15 (12.1%)	3 (3.8%)	0 (0.0%)	0.024
Device success	108 (87.1%)	74 (94.9%)	30 (100%)	0.031
PPI	21 (16.9%)	11 (14.1%)	5 (16.7%)	0.861
Stroke (including not disabling)	2 (1.6%)	2 (2.6%)	1 (3.3%)	0.806
30-day mortality	6 (4.8%)	4 (5.1%)	0 (0.0%)	0.458

PPI, permanent pacemaker implantation; PVL, paravalvular leak.

### 3.4 Predictors of Device Success and ≥Moderate PVL

Device success was achieved by 212 patients (91.4%).

On multivariate analysis, both annulus and LVOT calcifications (OR 0.105; *p* = 0.023) were independent predictors of device success (Table 7).

**Table 7.** **Univariate and multivariate predictors of device success**.

		Odds ratio	95% CI	*p*-value
Univariate predictors				
	Hypertension	1.903	0.739–4.900	0.182
	Dyslipidemia	2.839	0.918–8.871	0.070
	Ejection fraction (%)	0.956	0..907–1.008	0.099
	Mean aortic gradient (mmHg)	0.976	0.947–1.006	0.120
	LVOT diameter	1.210	0.976–1.500	0.082
	Calcification number	0.698	0.426–1.146	0.155
	Major calcification diameter (mm)	0.845	0.734–0.973	0.020
	Evolut-R	0.260	0.084–0.802	0.019
	Portico	2.145	0.692–6.650	0.186
	Implantation depth LCC (mm)	1.164	0.983–1.377	0.078
	Postdilatation	0.445	0.165–1.202	0.110
	Contrast volume (mL)	0.993	0.986–1.000	0.064
	LVOT Calcification	0.314	0.102–0.970	0.044
	Both Annulus and LVOT calcification	0.134	0.043–0.416	<0.001
Multivariate predictor				
	Both Annulus and LVOT calcification	0.105	0.015–0.736	0.023

LCC, left coronary cusp; LVOT, left ventricular outflow tract.

On univariate analysis, calcification location in LVOT, in both annulus and LVOT, use of an Evolut-R prosthesis and postdilatation were predictive of ≥moderate PVL (Table 8).

**Table 8.** **Univariate predictors of ≥moderate PVL**.

		Odds ratio	95% CI	*p*-value
Univariate predictors				
	Evolut-R	4.817	1.355–17.121	0.015
	Postdilatation	3.435	1.095–10.774	0.034
	Implantation depth LCC (mm)	0.857	0.717–1.023	0.088
	LVOT Calcification	3.992	1.123–14.193	0.032
	Both Annulus and LVOT calcification	9.267	2.600–33.030	<0.001

LCC, left coronary cusp; LVOT, left ventricular outflow tract.

### 3.5 Simulation Analysis

**Fig. 5.**
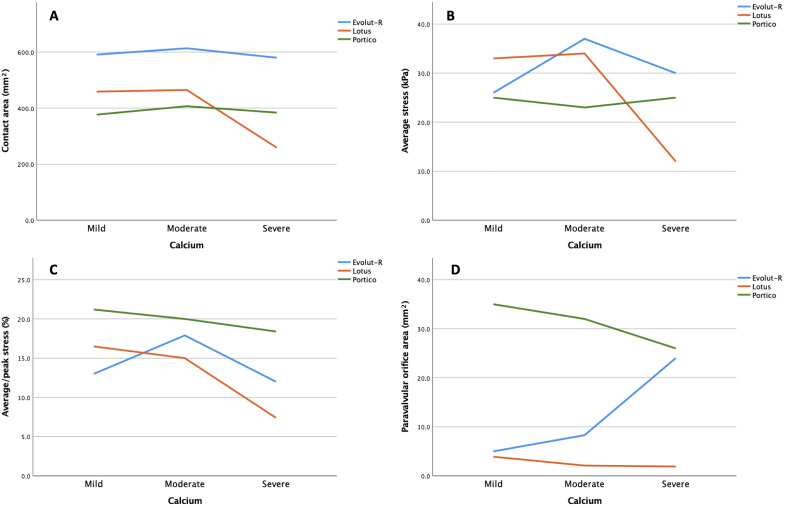
**Simulation analysis**. (A) Stent-root contact area, (B) average stress, (C) stress distribution and (D) paravalvular orifice area in relation to nodular calcific burden and valve type.

Results are reported in Fig. 5.

Evolut-R and Portico devices maintained similar values of stent-root interaction area independently from calcification burden, whereas Lotus showed a worsening in presence of severe calcifications (from 465 ${\mathrm{mm}}^{2}$ for moderate to 259 ${\mathrm{mm}}^{2}$ for severe). Similarly, Evolut-R and Portico exhibited comparable patterns of Von Mises stress distribution among the three models, whereas in the Lotus group there were no steep stress variations but a slight decrease in the presence of increased nodular calcification distribution. Evolut-R and Portico also showed comparable average stress in the three models, meanwhile Lotus showed a steep decrease in presence of severe calcifications.

As for PVL, Portico showed overall greater paravalvular orifice area for each grade of calcification (respectively 35 ${\mathrm{mm}}^{2}$ for mild, 32 ${\mathrm{mm}}^{2}$ for moderate, 26 ${\mathrm{mm}}^{2}$ for severe), as compared to Evolut-R (5 ${\mathrm{mm}}^{2}$ for mild, 8.3 ${\mathrm{mm}}^{2}$ for moderate, 24 ${\mathrm{mm}}^{2}$ for severe). On the other hand, the Lotus showed low values and no increases of paravalvular orifice area across the three models (3.9 ${\mathrm{mm}}^{2}$ for mild, 2.1 ${\mathrm{mm}}^{2}$ for moderate, 1.9 ${\mathrm{mm}}^{2}$ for severe) (Fig. 5).

## 4. Discussion

### 4.1 Study Findings

The present study showed that nodular calcifications in the aortic annulus and/or LVOT can be found in around 34% of patients undergoing TAVI. As expected, the presence of ≥moderate calcifications correlated with higher rates of ≥moderate PVL, longer procedural times, more vascular complications, and, ultimately, lower rate of device success compared to patients with mild calcifications. Among the three investigated devices, the Evolut-R showed a higher rate of ≥moderate PVL compared to the Portico and Lotus valve. The statistical significance of lower device success rate was reached in the Evolut group in comparison to the Lotus, but not to the Portico. Noteworthy, nodular calcifications in both aortic annulus and LVOT independently predicted device success.

On computational simulations, the Lotus valve, showed the best performance in each calcification scenario with respect to paravalvular orifice area, consistently with the clinical findings.

The Portico system exhibited an excellent stent conformability as stress distribution was substantially homogeneous by increasing calfications, but showed overall larger paravalvular orifice area apparently in contrast with the clinical data.

On the other hand, the Evolut-R showed higher values of stress on the aortic wall than Portico, although with a more dishomogeneous distribution in each calcification model; this finding resulted to greater paravalvular orifice areas by increasing calcifications, and it was confirmed in the clinical setting by a higher rate of ≥moderate PVL in presence of ≥moderate calcifications.

### 4.2 Literature Comparison 

The extent and distribution of calcifications has been under investigation by multiple studies which showed a correlation between LVOT tract involvement and PVL [8, 9, 25]. However, evidence is still modest since data were derived from observational studies. Mauri *et al*. [26] compared the performance of balloon expandable (BE), SE and ME valves, and found that the SE ones were more susceptible to PVL in presence of elevated calcium at the device landing zone. It has been hypothesized that SE prostheses have lower radial force than BE valves, mending to the shape imposed by the elliptic annulus [27], and as in our case by the calcific nodules. On the other hand, probably due to the higher radial force, the BE prostheses are also associated with increased risk of aortic root injury during TAVI procedures, especially in case of calcifications in the upper LVOT below the NCC [28]. However, these results are contrastating, as BE valves were found, in another report, to be independent predictors of device failure in presence of severe LVOT calcification [29].

### 4.3 Core Discussion 

The distribution of the stent stress is a key factor for the interection between nodular calcification and TAVI devices deployment. In fact, simulation findings showed that calcification dimension correlated directly with PVL area. Only the Lotus valve performed well in every setting due to its optimal radial force and conformability.

We tested these results in a real-world population who underwent Evolut-R, Portico and Lotus implantation. In the Lotus group simulation findings matched the clinical data, whereas this was not the case of Portico and Evolut-R in terms of paravalvular area. Notably, ≥moderate PVL rate was higher in Evolut-R patients (12.1%) compared to Portico (3.8%) with statistical significance (*p* = 0.045) despite similar calcium burden (*p* = 0.187).

Thus, when using SE valves in the presence of nodular calcifications, the sole stent evaluation is too unsophisticated, as additional aspects may be relevant to achieve optimal results.

Firstly, the conformability of the valve to the altered landing zone may be an alternative feature to the high radial force, especially if it does not reach the critical crushing force. The Portico design has larger cells than Evolut-R, translating into an homogeneous stress distribution by any level of calcification and ellipticity, and ultimately to an optimal stent conformability to the shape of aortic annulus.

Secondly, the simulations were conducted without considering predilatation and postdilatation: in our study Evolut-R group had a lower predilatation rate compared to Portico and Lotus (41.9% vs. 76.9% vs. 63.3% respectively, *p *< 0.001). Postdilatation was similar between Evolut-R (58.1%) and Portico (61.5%), meanwhile it was extremely rare in the Lotus group (6.7%). Therefore, predilatation with Portico is recommended to compensate its low radial force in order to alter the landing zone anatomy, thus allowing optimal valve expansion.

Thirdly, the Evolut-R could be more technically demanding because of its narrow landing zone (3–6 mm by manufacturer) and its flared stent design. In contrast, the Portico has a more tolerant implantation depth interval (1–9 mm by manufacturer). In this regard, in our study, 29.8% of patients treated with Evolut-R had a mean implantation depth >6 mm, whereas only 7.4% of patients treated with Portico had a mean implantation depth >9 mm. As further support, the Evolut-R was repositioned more frequently than the Portico (12.1% vs. 3.3%; *p *< 0.001). On the other hand, the use of Lotus device, being fully recapturable even after complete release, may explain the excellent device success and low PVL rate, observed in our study.

It has to be highlightened that calcifications both in the LVOT and annulus can give a higher underexpansion rather than a large calcification in a single zone, as showed by our multivariate analyses.

The clinical implications of our results are relevant as nodular calcifications can be observed in >30% of TAVR patients. We believe that a comprehensive assessment of strengths and pitfalls of new-generation devices (i.e., radial force, conformability, presence of external sealing skirt, implantation-related challenges) is mandatory to select the most appropriate device with respect to patient’s unique anatomy, in order to achieve procedural success.

### 4.4 Limitations

This study has several limitations. First, due to the retrospective design, device groups were not balanced, with Evolut-R being the predominant valve. As stated above this was due to our institutional policy.

Second, the distribution of calcifications location in the aortic annulus or LVOT were not comparable among the three devices. However, given that the device landing zone includes both annulus and LVOT, a single calcific nodule in this area of interest did not seem to be predictive of device success on multivariate analysis, whereas this was not the case if nodular calcifications in both regions were present.

Third, our study could not include patients treated with devices having an external sealing skirt such as Evolut Pro and Navitor valves which may have excellent outcomes in the setting of nodular calcifications.

As stated previously, the sealing skirt could not be integrated in the simulation model and our main focus was on the interaction between the stent struts and the aortic root.

Fourth, computational simulation could not take into account the impact of pre/postdilatation with a potential impact on simulation results. Similarly, on computational simulations, we had a simplified aortic root model with a single calcium nodule of increasing dimensions as marker of calcification severity. In a real-world setting, nodules can be multiple and of various dimensions. Finally, the results might be sensitive to the relative circumferential configuration between the calcium block and the stent mesh, which is more sparse in the case of Portico device than in the two other cases. Studies including patient-specific simulations are needed to further clarify the interaction between TAVI devices and aortic annuli with complex calcium distribution.

## 5. Conclusions

At least moderate nodular calcifications significantly impacted TAVI outcome, especially in presence of nodules located both in LVOT and aortic annulus. Among the investigated devices, Lotus and Portico seemed to perform better than Evolut-R as for device success and ≥moderate PVL.

On computational simulation the three devices exhibited unique biomechanical features in terms of force and conformability of the stent frame with respect to calcifications size.

Therefore, a comprehensive assessment of both device features and aortic annulus/LVOT anatomy is pivotal in order to achieve optimal procedural outcome in these common although complex patients.

## Abbreviations

AMI, acute myocardial infarction; AR, aortic regurgitation; CABG, coronary artery bypass grafting; COPD, chronic obstructive pulmonary disease; CT, computed tomography; IE, index of eccentricity; HU, Hounsfield Units; LCC, left-coronary cusp; LM, left main; LVOT, left ventricular outflow tract; ME, mechanically-expandable; NCC, non-coronary cusp; PPI, permanent pacemaker implantation; PVL, paravalvular leak; RCA, right coronary artery; SE, self-expanding; STS, Society of Thoracic Surgeons; TAVI, trancatheter aortic valve implantation; THV, transcatheter heart valve; TTE, transthoracic echocardiography; VARC-3, Valve Academic Research Consortium-3.
